# Case Series: Acute Hemorrhagic Encephalomyelitis After SARS-CoV-2 Vaccination

**DOI:** 10.3389/fneur.2021.820049

**Published:** 2022-02-02

**Authors:** Mihai Ancau, Friederike Liesche-Starnecker, Johanna Niederschweiberer, Sandro M. Krieg, Claus Zimmer, Charlotte Lingg, Daniela Kumpfmüller, Benno Ikenberg, Markus Ploner, Bernhard Hemmer, Silke Wunderlich, Mark Mühlau, Benjamin Knier

**Affiliations:** ^1^Department of Neurology, Klinikum Rechts der Isar, Technical University of Munich, Munich, Germany; ^2^Department of Neuropathology, Institute of Pathology, Technical University of Munich, Munich, Germany; ^3^Department of Neurosurgery, Klinikum Rechts der Isar, Technical University of Munich, Munich, Germany; ^4^Department of Neuroradiology, Klinikum Rechts der Isar, Technical University of Munich, Munich, Germany; ^5^Department of Anesthesiology and Intensive Care Medicine, Klinikum rechts der Isar, Technical University of Munich, Munich, Germany; ^6^Munich Cluster for Systems Neurology (SyNergy), Munich, Germany

**Keywords:** acute hemorrhagic encephalomyelitis, acute disseminated encephalomyelitis, acute hemorrhagic leukoencephalitis, Weston-Hurst syndrome, vaccination, ChAdOx1 nCoV-19, COVID-19

## Abstract

We present three cases fulfilling diagnostic criteria of hemorrhagic variants of acute disseminated encephalomyelitis (acute hemorrhagic encephalomyelitis, AHEM) occurring within 9 days after the first shot of ChAdOx1 nCoV-19. AHEM was diagnosed using magnetic resonance imaging, cerebrospinal fluid analysis and brain biopsy in one case. The close temporal association with the vaccination, the immune-related nature of the disease as well as the lack of other canonical precipitating factors suggested that AHEM was a vaccine-related adverse effect. We believe that AHEM might reflect a novel COVID-19 vaccine-related adverse event for which physicians should be vigilant and sensitized.

## Introduction

In the context of the current coronavirus disease 2019 pandemic (COVID-19), the non-replicating vector vaccine ChAdOx1 nCoV-19 (AstraZeneca, UK) is one of the most frequently employed vaccines worldwide. The mass public immunization program against COVID-19 in Germany ensued in December 2020. Several side effects including demyelinating disorders of the central nervous system (1–3), Guillain-Barré syndrome (GBS) (4, 5) and vaccine-induced immune thrombotic thrombocytopenia (VITT) (6) have been reported. On the one hand, isolated cases of potential acute disseminated encephalomyelitis, ADEM, which is a rare autoimmune event potentially occurring after an immunological challenge, such as a vaccination, have been linked to COVID-19 vaccines (7–11). On the other hand, COVID-19 itself has been associated with cases of hyperacute hemorrhagic variants of ADEM (acute hemorrhagic encephalomyelitis, AHEM, Weston-Hurst syndrome or acute hemorrhagic leukoencephalitis, AHLE) (12–16). We now report three cases of AHEM occurring in the context of COVID-19 vaccination, namely within 9 days after the first shot of ChAdOx1 nCoV-19. None of the cases exhibited a cerebral venous thrombosis. Written informed consent was obtained from all patients or their legal representatives.

## Case 1

**Figure 1:**
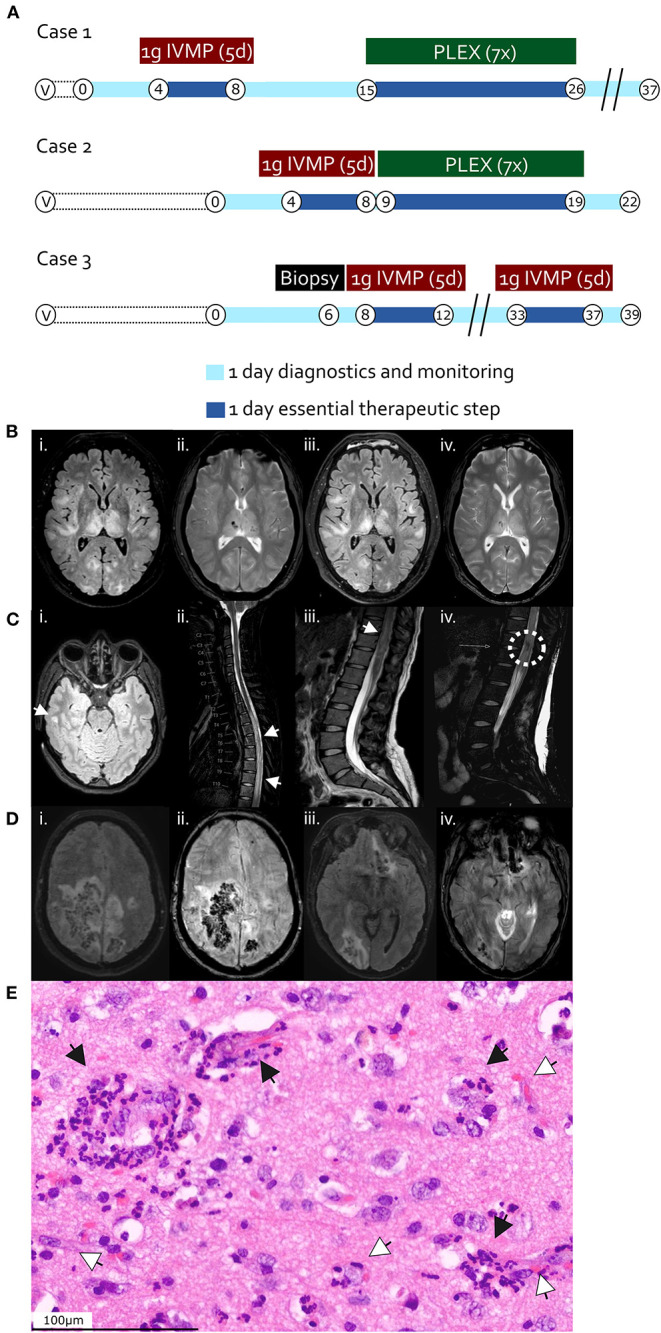
**(A)** Time course and therapy in case 1 (first row), case 2 (second row), and case 3 (third row). “V”, vaccination timepoint; “B”, Biopsy and hemicraniectomy timepoint in case 3; “IVMP”, intravenous methylprednisolone; “PLEX”, Plasma exchange. Encircled numbers indicate days after admission. **(B–D)** Magnetic resonance imaging (MRI) findings. **(B)** Case 1, day 2 after admission; brain axial T2 FLAIR (i) and T2 haem-sensitive sequence (ii); follow-up brain MRI at day 7 after admission, after methylprednisolone pulse regimen; brain axial T2 FLAIR (iii) and T2 haem-sensitive sequence (iv). **(C)** Case 2, day 1 after admission; brain axial T2-FLAIR (i); sagittal cervico-thoracic spinal T2 (ii); sagittal thoraco-lumbar spinal T2 (iii, iv). White arrowheads indicate FLAIR-hyperintense lesions (i–iii), the dashed white circle in iv highlights a region of spinal hemorrhage. **(D)** Case 3, day 2 after admission; brain axial T2 FLAIR (i, iii) and SWI (ii, iv) sequences. **(E)** Hematoxylin and eosin-stained section from right temporal cortical biopsy of case 3 (day 6), displaying perivascular immune cell infiltrates including neutrophilic granulocytes (black arrow heads), tissue edema, and perivascular hemorrhage (white arrow heads). Scale bar 100 μm.

A 61-year-old male with a history of hypothyroidism and polymyalgia rheumatica developed fever, headache and apathy 2 days after the first shot of the ChAdOx1 nCoV-19 vaccine. Two days later, he was discovered by his wife unconscious in his bed foaming around the mouth. Upon arrival of the emergency doctor, he exhibited a generalized seizure, was subsequently comatose and underwent endotracheal intubation. A head CT scan including CT-angiography revealed diffuse hypodense areas in the right subcortical frontotemporal and the right thalamic region. There were no signs of vessel occlusions, especially no sinus vein thrombosis. Magnetic resonance imaging (MRI) revealed bilateral confluent cortical and subcortical FLAIR hyperintense lesions with hemorrhagic involvement of the basal ganglia (Figure 1B). Cerebrospinal fluid (CSF) examination revealed normal cell counts (1 leukocyte per μl) and moderate disturbance of the blood-brain-barrier [CSF/serum quotient for albumin of 22.8 × 10^−3^, age-adjusted upper reference limit 10.2 × 10^−3^ (17)]. No CSF-specific oligoclonal bands or intrathecal IgG/-A/-M-synthesis were detected, while flow cytometry analysis revealed normal CSF cell subsets (64% CD4^+^ T cells, 19% CD8^+^ T cells, 7% CD14^+^ monocytes, 0.7% CD19^+^ B cells, and 4% CD56^+^ NK cells as portions of CD45^+^ cells in the CSF). Laboratory testing for bacterial and viral infectious agents of the central nervous system *via* serology (in serum) and polymerase chain reaction (CSF and/or serum) remained negative, including testing for Human immunodeficiency virus, Borrelia burgdorferi, Treponema pallidum, Herpes simplex virus, John Cunningham virus, tick-borne encephalitis virus, Epstein-Barr virus, measles, mumps, rubella West Nile/lymphocytic choriomeningitis-/varicella-zoster virus and SARS-CoV-2 repeatedly *via* RT-PCR from nasopharyngeal swabs. We did not find abnormal antibody titers against aquaporin-4 (AQP4) or myelin oligodendroyte glycoprotein (MOG) in cell-based assays (CBA). Screening for antinuclear antibodies (ANA), antineutrophil cytoplasmic antibodies (ANCA), antiphospholipid antibodies, neuronal and paraneoplastic antibodies were negative.

We assumed an episode of AHEM and administered high dose steroid treatment (1 g methylprednisolone intravenously per day) over 5 days, followed by seven plasma exchange sessions (Figure 1A) with concomitant methylprednisolone administration (250 mg per day *via* nasogastric tube on days of plasma exchange, 100 mg per day *via* nasogastric tube on days between plasma exchanges, followed by tapering beginning with 100 mg orally per day and subtraction of 20 mg every 2 days), upon which there was slight improvement of alertness and reduction in size of the brain lesions in follow-up MRI already after 5 days. On clinical follow-up after 14 weeks of rehabilitation, the patient presented with a vegetative state.

## Case 2

A 25-year-old woman without medical record developed severe cephalgia, thoracic back pain, mild weakness and ascending numbness in her legs 9 days after the first shot of the ChAdOx1 nCoV-19 vaccine. The symptoms evolved over the course of one and a half days to a complete paraplegic syndrome including anesthesia below dermatome T6, absent tendon reflexes of the lower extremities and detrusor areflexia with urinary retention. Spinal MRI revealed a longitudinal edema throughout the thoracic spinal cord exhibiting mild contrast enhancement as well as focal central hemorrhages (Figure 1C). Cranial MRI showed bi-hemispheric white matter lesions with focal contrast enhancement. CSF examination showed granulocytic pleocytosis (initially on automatic counting, including erythrocytes, 5,284 cells/μl, on repeated tap and manual counting 241 leukocytes/μl, predominantly granulocytes) and a highly elevated CSF/serum quotient for albumin of 164.7 × 10^−3^ [age-adjusted upper reference limit 6.8 × 10^−3^ (17)]. No CSF-specific oligoclonal bands were detected. Intrathecal IgM synthesis, but not IgG or IgA synthesis was detected, while flow cytometry analysis revealed a mildly increased B cell proportion among lymphocytic populations in pink-tinged CSF (62% CD4+ T cells, 12% CD8+ T cells, 6% CD14+ monocytes, 1.7% CD19+ B cells, and 2% CD56+ NK cells). Laboratory testing for bacterial and viral infectious agents *via* serology (in serum) and PCR (in CSF and/or serum), including neurotropic viruses, meningitis bacteria, Mycobacterium tuberculosis, toxoplasmosis, bartonellosis, brucellosis, Treponema pallidum, Borrelia burgdorferi, leptospirosis, Human immunodeficiency virus and tick-borne encephalitis virus serology were negative. Moreover, glial-, neuronal-targeting, and paraneoplastic autoantibodies (CBA for AQP4- and MOG-, immunofluorescence assays in the serum for ANA, ANCA, anti-double stranded DNA antibodies) proved negative. SARS-CoV-2-RT-PCRs from nasopharyngeal swabs were also repeatedly negative.

Considering an autoimmune acute longitudinal extensive transverse and hemorrhagic myelitis (spinal variant of AHEM), the patient was put on a high dose steroid treatment (1 g methylprednisolone intravenously per day over 5 days), upon which the cephalgia improved drastically and the sensory components slightly. The therapy was followed by seven plasma exchange sessions (Figure 1A) with concomitant methylprednisolone administration (250 mg per day orally on days of plasma exchange, 100 mg per day orally on days between plasma exchanges) and subsequent methylprednisolone tapering over 10 days beginning with 100 mg orally per day after the last plasma exchange and subtraction of 20 mg every 2 days, leading to clinical improvement of only sensory symptoms but persistent paraplegia on 6-week follow-up.

## Case 3

A 55-year-old woman developed progressive nausea, dizziness and meningism 9 days after the first shot of the ChAdOx1 nCoV-19 vaccine. Symptoms worsened rapidly to severe spastic tetraparesis and coma. Brain MRI revealed multiple FLAIR-hyperintense and hemorrhagic lesions in the right parietal and temporal lobes, bilaterally in fronto-temporal distribution as well as in the right occipital lobe and left fronto-basal region (Figure 1D). There were no signs of cerebral sinus vein thrombosis. CSF showed mixed granulocytic and lymphocytic pleocytosis (10/μl) and a normal CSF/serum quotient for albumin of 7.4 × 10^−3^ [age-adjusted upper reference limit 10.2 × 10^−3^ (17)]. No CSF-specific oligoclonal bands were detected. Intrathecal IgM, IgA and IgG synthesis was detected, while flow cytometry analysis revealed an increased B cell proportion among lymphocytic populations (56% CD4+ T cells, 27% CD8+ T cells, 5% CD14+ monocytes, 6% CD19+ B cells, and 1% CD56+ NK cells). Laboratory testing for infectious agents *via* serology (in serum) and PCR (in CSF and/or serum), including cytomegalovirus, herpes simplex virus, varicella-zoster virus, tick-borne encephalitis virus, enteroviruses, Borna disease virus, West Nile Virus, Sandfly Fever Naples-Virus, human immunodeficiency virus, Treponema pallidum, Bartonella henselae, Brucella melitensis, Coxiella burnetii, Acanthamoeba, Toscana virus, Leptospirosis, Borrelia burgdorderi, Ehrlichia, Rickettsia, Babesia, Naegleria fowleri proved negative. Both autoimmune (AQP4-, MOG-autoantibodies as measured by CBA), and paraneoplastic antibodies (immunofluorescence assays in the serum) were negative. SARS-CoV-2-RT-PCRs from nasopharyngeal swabs were repeatedly negative as well. She developed increased intracerebral pressures and became comatose while developing anisocoria with right non-reactive mydriasis. Subsequent imaging a revealed 11 mm midline shift to the left, manifest trans-tentorial herniation and hydrocephalus occlusus. She underwent emergency right-sided decompressive hemicraniectomy. A brain cortex biopsy from the affected right temporal lobe revealed perivascular predominantly granulocytic infiltrates and hemorrhages (Figure 1E). Conventional as well as computed tomography angiography were unremarkable without signs of vasculitis.

The patient was put on a high dose steroid treatment (1 g methylprednisolone intravenously per day over 5 days) with subsequent tapering over 10 days (beginning with 100 mg *via* nasogastric tube per day and subtracting 20 mg every 2 days), which led to significant improvement of vigilance and motor function (Figure 1A). Two weeks after steroid therapy, her state worsened again due to new brainstem and occipital FLAIR-hyperintense and hemorrhagic lesions. A repeat high dose steroid treatment remained without positive effects and the patient died due to progressive intracerebral hemorrhage of the brain stem. An autopsy was declined by members of the family of the patient.

## Discussion

In this report, we describe three patients who developed acute demyelinating and hemorrhagic lesions of the central nervous system consistent with AHEM within 9 days of the first dose of the ChAdOx1 nCoV-19 vaccine during a 7-week period between May and June 2021. ADEM constitutes a rare demyelinating disease of the brain and spinal cord of mostly monophasic nature, first described toward the end of the eighteenth century following measles infection and smallpox vaccinations (18). It is a relatively seldom neurological illness, which can occur at any age, but predominates in children and young adults, amounting to an incidence of around 0.4 cases per 100,000 people per year.

Hyperacute hemorrhagic variants of ADEM, also known as AHEM, have firstly been described by Hurst in 1941 (19). Deducing from the very limited number of AHEM cases reported worldwide, they represent up to about 2% of ADEM cases (therefore around 0.008 cases per 100,000 people per year) (20), and exhibit a fulminant clinical presentation, comprising fever, meningoencephalopathic features, seizures and a rapid progression to coma in addition to the hallmarks of ADEM (18). Imaging reveals large white matter lesions with parenchymal edema accompanied by punctual or confluent hemorrhages (21). In contrast to ADEM, prognosis is mostly poor despite timely steroid treatment or plasma exchange (18, 21, 22). Histopathological examination reveals perivascular immune cell infiltrates including neutrophilic granulocytes, pronounced tissue edema and perivascular hemorrhage, as shown in case 3 (21–24).

The considerable variation in CSF cell numbers (case 1 – normal cell count; case 2 – >5,000/μl including blood cells, 241/μl leukocytes; case 3 – 10/μl) was not incompatible with previous reports of AHEM/AHLE in the literature, one recent review indicating CSF cell number ranges of up to 2,100/μl, but mostly up to 360/μl (mixed mono- and polymorphonuclear) (21).

The patients in the presented cases developed neurological symptoms between 2 and 9 days after the first vaccination and fulfilled the diagnostic criteria set by the International Pediatric MS Study Group (25) and the Brighton Collaboration Encephalitis Working Group (26) for ADEM. Evidence-based diagnostic criteria for AHEM have due to the rarity of the disease not yet been defined, but consensus definitions exist (18, 21). In accordance with the latter, additional clinical features like meningitic syndromes, radiological findings with multifocal hemorrhages (including the spinal cord), histopathological findings and the fulminant progression to encephalopathy suggested the rare variant AHEM in all three cases.

Reports of potential vaccine-related adverse effects from the European Medicines Agency's (EMA) *EudraVigilance* database (27) revealed slightly higher, but altogether comparable incidences of 0.07 (ChAdOx1 nCoV-19) and 0.05 (Ad26.COV2.S) per 100,000 people per year for potential vector-based vaccine associated ADEM, compared to 0.02 (BNT162b2) and 0.04 (mRNA-1273) for mRNA-vaccines (Table 1). Concerning AHEM, only one other report after COVID-19 vaccination was found in the *EudraVigilance* database, after inoculation with ChAdOx1 nCoV-19. Studies using the *Vaccine Safety Datalink* have reported a possible association between ADEM and vaccination (especially influenza vaccines) (20) with an incidence of 0.1–0.2 per 100,000 (28). However, even considering the present cases in addition to the single report from *EudraVigilance*, the incidence of potentially ChAdOx1 nCoV-19 associated AHEM (0.006 per 100,000 per year) would not exceed the vaccine-independent incidence (see above, 0.008 per 100,000 per year) (Table 1).

**Table 1:** Reported number of potential vaccine-related adverse effects in European Economic Area (EEA) countries associated with the terms “acute disseminated encephalomyelitis” or “acute haemorrhagic leukoencephalitis” in the European Medicines Agency's (EMA) *EudraVigilance* database by 20th November 2021.

**Vaccine name**	**ChAdOx1 nCoV-19 (AstraZeneca–University of Oxford)**	**BNT162b2 (Pfizer–BioNTech)**	**mRNA-1273 (Moderna)**	**Ad26.COV2.S (Johnson & Johnson)**
No. of ADEM cases	46	91	27	8
No. of AHEM cases	1 (+3)	0	0	0
No. of vaccine doses administered	68,835,033	453,805,251	63,971,912	17,477,802
Derived incidence of potential vaccine associated ADEM	0.067	0.020	0.042	0.046
Derived incidence of potential vaccine associated AHEM	0.0015 (0.006)	-	-	-

*Total number of vaccine doses administered as of 19th November 2021 according to the European Center for Disease Prevention and Control (ECDC). Incidence values quantified per 100,000 people per year. In case of AHEM, results without and with the current cases (numbers in brackets) presented. EEA population: 519,832,354*.

The cumulative occurrence of a series of rare variants of an uncommon disease in the space of a couple of months was remarkable and achieved a score of 6 (“probable”) for the association with the vaccination on the Naranjo Adverse Drug Reaction Probability Scale (29). This score on the scale suggested that there was indeed a true association, possibly even a causal link between the ChAdOx1 nCoV-19 vaccine and AHEM, and that the occurrences were not purely coincidental. In this context, however, given the high numbers of people already vaccinated against COVID-19, a fortuitous link between the inoculation event and a neurological disorder occurring by chance in the post-vaccination window is also probable. The short time span from inoculation to the onset of neurological symptoms is common for vaccine associated ADEM/AHEM, which may occur between 1 and 30 days after inoculation with non-neurotropic vaccines (30, 31). The accumulation of these 3 AHEM cases between the beginning of May to end of June 2021 coincided with a near doubling in the number of vaccinations with ChAdOx1 nCoV-19 during the months April to May 2021 in the Federal Republic of Germany (32). Also, by the beginning of June the first reports of VITT and of GBS as potential vaccine-triggered adverse reactions after vaccination with ChAdOx1 nCoV-19 had emerged, so that in light of increased general awareness the detection of other potential neurological complications in the post-vaccination window also became more probable. Considering the current population of Munich and its area of influence as well as presuming that AHEM events follow a Poisson distribution with an average rate of occurrence equal to the incidence of vaccine-associated AHEM (independent of vaccine type, see above), the observed regional accumulation of three cases in the given time span would have a not inconsiderable probability of occurrence between 0.3 and 7%.

A pathophysiologic link between vaccination and AHEM may be given by molecular mimicry (shared pathogenic epitopes between infectious agent/vaccine and molecular central nervous structures) or a re-infectious etiology (opening of the blood-brain-barrier and breakdown of immune tolerance by a preceding CNS infection) (31). ChAdOx vaccines have been proven to elicit particularly strong T cell responses (33), biased toward IFNγ secretion and a Th1-phenotype. Even though both the mRNA and vector-based vaccines encode production of the spike (S) protein of SARS-CoV-2, they differ with respect to the adjuvant required to stimulate the innate immune system: for mRNA vaccines, the mRNA itself acts as both immunogen and adjuvant, due to its recognition by endosomal and cytosolic innate sensors (i.e., TLR3, TLR7), whereas in the case of vector-based vaccines it is elements of the virus particle that are recognized by pattern recognition receptors (i.e., TLR9) (34).

In any case, we cautiously surmise that these cases demonstrate severe and fulminant AHEM and suggest physicians should be vigilant in recognizing such cases in patients who have received the ChAdOx1 nCoV-19, while vaccine surveillance programs could be sensitized to capture data on this potentially devastating outcome. The temporal relationship, however, between vaccination and AHEM alone is not sufficient to prove causality. Millions of people have received COVID-19 vaccines by now, and therefore the chance of neurological conditions occurring within the post-vaccination window by chance alone is also considerable. Even if the reported cases are indeed vaccine-associated, the benefits of vaccination in reducing morbidity and mortality still by far outweigh the risks of ongoing vaccination programs.

## Data Availability Statement

The original contributions presented in the study are included in the article/supplementary material, further inquiries can be directed to the corresponding author/s.

## Ethics Statement

Ethical review and approval was not required for the study on human participants in accordance with the local legislation and institutional requirements. The patients/participants provided their written informed consent to participate in this study. Written informed consent was obtained from the individual(s) for the publication of any potentially identifiable images or data included in this article.

## Author Contributions

MA, MM, and BK contributed to the study concept and design. MA, FL-S, JN, SK, CZ, CL, DK, BI, MP, BH, SW, MM, and BK participated in the data acquisition and analysis. MA and BK wrote the manuscript with contributions from all co-authors. All authors contributed to the article and approved the submitted version.

## Conflict of Interest

The authors declare that the research was conducted in the absence of any commercial or financial relationships that could be construed as a potential conflict of interest.

## Publisher's Note

All claims expressed in this article are solely those of the authors and do not necessarily represent those of their affiliated organizations, or those of the publisher, the editors and the reviewers. Any product that may be evaluated in this article, or claim that may be made by its manufacturer, is not guaranteed or endorsed by the publisher.
